# Biovalorization of Nondetoxified Corn Cob Hydrolysate into Xylitol by *Pichia fermentans* T12

**DOI:** 10.1021/acsomega.6c00635

**Published:** 2026-06-18

**Authors:** Mehmet Akif Omeroglu

**Affiliations:** Department of Molecular Biology and Genetics, Faculty of Science, 37503 Atatürk University, Erzurum 25240, Turkey

## Abstract

Xylitol is a sugar alcohol extensively used in the food and pharmaceutical industries and can be produced via microbial fermentation processes through the reduction of xylose derived from hemicellulosic biomass. Corn cobs represent an abundant lignocellulosic resource that can serve as a raw material for xylitol production as well as the biosynthesis of other value-added compounds. The objective of the current study was to examine the novel utilization of corn cob hydrolysate (CCH), rich in xylose, as a sustainable and low-cost substrate for xylitol production by the newly isolated yeast strain *Pichia fermentans* T12 (GenBank number: PV876729). Acid-pretreated corn cobs, without any detoxification step, were directly employed as the feedstock for xylitol production. The process parameters were optimized to enhance the xylitol yield, and the highest xylitol accumulation was achieved at a temperature of 30 °C, an initial pH of 6, a CCH concentration of 125 mL/L, a yeast extract concentration of 2 g/L, an inoculum concentration of 7%, and an incubation time of 96 h. Under optimized batch fermentation conditions using 125 mL/L CCH (32 g/L xylose), 21.6 g/L xylitol was obtained with a yield of 0.67 g/g, as confirmed by the HPLC analysis. Following downstream processing, the functional groups of the microbial xylitol were verified by FTIR results. This research study emphasizes the potential of *P. fermentans* T12 for xylitol production by utilizing corn cob biomass as a microbial cell factory, offering a sustainable alternative to conventional substrates.

## Graphical abstract

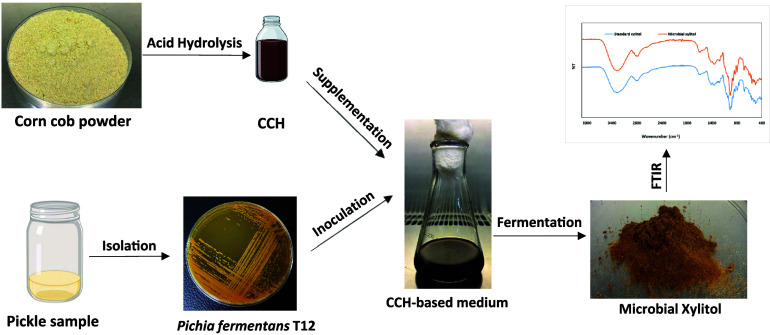

## Introduction

Producing commodity chemicals from renewable sources is considered one of the most environmentally friendly alternatives to petroleum-based resources, which are major contributors to climate change and global warming.^1^ Biotechnologically important products derived from lignocellulosic biomass are gaining significant interest among researchers due to their potential as sustainable alternatives to fossil fuels, particularly in the context of waste management and efficient utilization of resources.^2,3^ The use of agricultural residues for industrial applications is drawing attention as a renewable source of raw materials instead of petroleum-derived raw materials. Lignocellulosic biomass is abundant in nature, cost-effective, renewable, and environmentally friendly. These byproducts, originating from agricultural and forestry waste, serve as valuable feedstocks for microbial fermentation processes used in the production of value-added biochemicals.^4,5^

Among the various applications of hexose and pentose sugars found in lignocellulosic biomass, xylitol production has been extensively studied.^6–8^ Xylitol, a five-carbon sugar alcohol, has recently gained significant attention in several industrial sectors. Xylitol has numerous applications in the food and pharmaceutical industries due to its diabetic-friendly sweetness, low caloric content, anticariogenic effects, and antimicrobial properties.^9,10^ Additionally, xylitol is recognized as one of the 12 key high-value intermediates, as it serves as a fundamental building block for the synthesis of various basic chemicals. Conventional xylitol production involves the chemical hydrogenation of xylose using a nickel catalyst at high temperatures and pressures. However, the chemical method has several drawbacks, including high energy demands, wastewater pollution, and the need for complex purification steps.^11,12^ As an alternative approach to chemical processes, xylitol production can be performed via microbial fermentation processes, which are more eco-friendly and cost-effective. Bioconversion of xylose into xylitol by microorganisms is based on the xylose reductase enzyme, which reduces xylose to xylitol using NADPH as a coenzyme. Various microbial strains have been investigated for their ability to convert hemicellulosic biomass into xylitol under suitable conditions. Yeast species belonging to the genera *Candida*, *Pichia*, *Debaryomyces*, *Kluyveromyces*, and *Spathaspora* have been reported to be the most efficient xylitol producers among microbes.^13,14^ *Pichia fermentans*, a mesophilic yeast, is considered to be a promising candidate for xylitol bioproduction because of its status as “generally regarded as safe” (GRAS). Several studies revealed that xylose-utilizing yeast *P. fermentans* has the ability to produce xylitol using agro-industrial residues.^15–19^

Corn cob, an abundant lignocellulosic byproduct of corn farming or processing industries, serves as an excellent feedstock for microbial xylitol production due to its high hemicellulose content (35–45%), composed primarily of xylose.^20^ Typically, corn cobs are dried and burned as firewood for household energy, leading to environmental issues like air, soil, and land pollution. This agricultural waste can be utilized as a nutrient-rich substrate to sustain microbial growth for the production of valuable industrial compounds.^21–23^ Global maize production ranks among the highest of all agricultural commodities, with annual grain yields exceeding approximately 1.2 billion metric tons. This large-scale production generates substantial amounts of agricultural residues, particularly corn cobs, which account for roughly 18 kg per 100 kg of harvested grain.^24^ Since 1978, xylitol production has expanded more than 40-fold, reaching a combined annual output of about 6000 t of xylitol and mannitol. The market price of xylitol typically ranges between USD 2.5 and USD 10 per kilogram, depending on factors such as geographic region and raw material source. Projections indicate that prices are likely to stabilize at around USD 4–5 per kilogram in the upcoming years.^25^ In this context, xylose-rich corn cob can be effectively utilized by microorganisms in the xylitol production. The current study focused on the utilization of corn cob in the microbial production of xylitol. For this purpose, xylose-assimilating yeast isolates were screened for their ability to grow on corn cob biomass and produce xylitol. The best xylitol-producing strain was identified as *P. fermentans* T12, and process parameters were optimized in order to enhance xylitol production. Despite numerous studies on xylitol production by *P. fermentans* strains, no research to date has focused on utilizing corn cob biomass.^15,16,18,19^ To the best of our knowledge, this is the first study on the bioconversion of corn cob into xylitol by *P. fermentans*.

## Materials and Methods

### Preparation of Corn Cob Hydrolysate (CCH)

Corn cob biomass, utilized as the source of xylose for xylitol production, was selected as the raw material and procured from local farmlands situated in Erzurum, Turkey. The biomass was washed properly with distilled water to remove impurities and dried in an oven at 80 °C. The dried samples were then ground into fine particles. Corn cob powder was subjected to acid hydrolysis using 1% (v/v) H_2_SO_4_ at 110 °C with a solid-to-liquid ratio of 1:10 (w/v) for 3 h. Following hydrolysis, the solids were removed by filtration, and the resulting filtrate was initially adjusted to pH 10 using calcium oxide, followed by neutralization to pH 6 with phosphoric acid. Vacuum evaporation was employed to concentrate the hydrolysate and prevent the thermal degradation of sugars. The hydrolysate was stored at 4 °C overnight and filtered again to remove the precipitated Ca_3_(PO_4_)_2_.^26,27^

### Isolation of Xylose-Assimilating Yeasts

Naturally fermented pickle samples (pickled cucumber, carrot, and garlic) were used as an isolation source for yeast strains. For this purpose, 1 mL of each collected sample was serially diluted (10^–1^-10^–6^) with sterile physiological water (0.9% NaCl), and 100 μL of each dilution was then spread on YPX agar plates consisting of 10 g/L yeast extract, 20 g/L peptone, 20 g/L xylose, and 20 g/L agar.^28^ The pH of the medium was initially adjusted to 5.5, and bacterial growth was prevented by adding chloramphenicol 0.01% (w/v). After the plates were incubated at 28 °C for 24–48 h, the morphological characteristics of the fungal colonies developing on the plates were examined by using an Olympus BX51 microscope. The yeast isolates showing different morphologies were subcultured and purified.^29^

### Screening of Xylitol Production Potential in Xylose-Assimilating Yeast Isolates

Screening experiments to evaluate the xylitol production abilities of yeast isolates were performed in 250 mL Erlenmeyer Flasks containing 100 mL of YPX liquid medium. Seed cultures of each isolate were prepared in Potato Dextrose Broth (PDB) medium by incubating at 30 °C in a shaking incubator set to 200 rpm. Afterward, 1 mL of the seed culture was inoculated into 100 mL of fermentation medium and incubated at 30 °C. After 48 h of fermentation, the cell-free supernatant obtained after centrifugation was analyzed in order to determine the xylitol concentration.^19^ The isolate T12, which had the highest xylitol accumulation, was used for subsequent experiments.

### Molecular Identification of the Best Xylitol-Producing Isolate

Total genomic DNA of the strain T12 was isolated using the Promega Wizard Genomic DNA Purification Kit. The ITS region, which is crucial for the fungal systematics, was amplified under in vitro conditions using universal primers ITS1 (5′-TCCGTAGGTGAACCTGCGG-3′) and ITS4 (5′-TCCTCCGCTTATTGATATGC-3′). Following PCR amplification, it was cloned into the pGEM-T Easy Vector System (Promega). Plasmid DNA was then isolated using the Promega (A1330) isolation kit and sent to Macrogen (Netherlands) for sequence analysis. The resulting sequence data obtained from the company were processed and analyzed using BioEdit software.^30^

### Optimization of Culture Conditions for Xylitol Production

In order to enhance xylitol production, several culture parameters, such as substrate concentration, temperature, initial pH, inoculum rate, and incubation time, were assessed through one-variable-at-a-time (OVAT) analysis. Preliminary experiments were focused on detecting the optimum temperature (26–34 °C) and initial pH (5–7) for xylitol production. Afterward, the optimal concentrations of CCH and yeast extract were determined. For this purpose, xylose-rich CCH was supplemented to the production medium at varying concentrations (50–150 mL/L). Correlatively, the effect of yeast extract (0.5–2.5 g/L) on xylitol production was evaluated. Ultimately, the effects of different inoculum rates (1–9%) and various incubation times (24–120 h) were tested. All fermentation experiments were performed in 250 mL flasks containing 100 mL of the production medium under shaking conditions.

### Analytical Methods

After fermentation, the culture medium was centrifuged at 4000*g* and 4 °C for 10 min to remove yeast cells. The cell-free supernatant was then treated with 4% activated charcoal at 200 rpm for 1 h to clarify, which effectively removed colorants and impurities. The liquid solution obtained after the treatment was evaporated by a vacuum evaporator at 40 °C. Afterward, the concentrated solution was blended with 50% (v v^–1^) ethanol and cooled at 5 °C. Following the addition of 1 g/L of commercial xylose in order to enhance the nucleation rate, the mixture was incubated at −20 °C for 48 h. The resulting crystals were filtered, washed with water to remove residual liquid, and placed in Petri dishes to dry at room temperature for 24 h.^31^

Xylitol analysis was carried out using Megazyme D-sorbitol/xylitol Assay Kit (Megazyme, Wicklow, Ireland). Xylose and xylitol concentrations were measured by High Performance Liquid Chromatography (Shimadzu Prominence) equipped with a refractive index detector at 40 °C and an Inertsil NH2 analytical column. The mobile phase consisted of acetonitrile and water in a 75:25 (v/v) ratio, flowing at 1.0 mL/min, and the column temperature was maintained at 30 °C. An injection volume of 20 μL was used for both standards.^32^

### Statistical Analysis

Each experiment was performed at least three times. Analysis of variance was carried out according to the one-way ANOVA test using Prism software 7.0 (GraphPad Software, San Diego, CA), and the averages were compared with the Duncan test at a confidence level of 0.05.

## Results and Discussion

### Characterization of Corn Cob Hydrolysate (CCH)

Characterizing lignocellulosic waste is crucial for its utilization as feedstock in biorefineries and bioprocesses. Determining their structural components allows for better optimization of their conversion into fermentable sugars in the efficient production of fuels, pharmaceuticals, fine chemicals, and other value-added products.^33,34^ Lignocellulosic materials are mainly composed of cellulose (35–50%), hemicellulose (20–35%), and lignin (5–30%). Nevertheless, the relative proportions of these components can vary depending on factors such as origin, species, and cultivation conditions.^35^ CCH is a promising source of lignocellulosic biomass, characterized by a high hemicellulose content, with xylose reported as its predominant sugar. In microbial fermentation processes, the use of nondetoxified hydrolysates offers several advantages, such as reduced process cost, simpler operation, and avoidance of sugar loss during detoxification; however, it comes with some drawbacks, such as fermentation inhibition caused by toxic compounds like furfurals and phenolics, reducing microbial growth and product yield.^36–38^ The xylose characterization of nondetoxified CCH performed in the current study showed that concentrated CCH contains 256 g/L of xylose; however, the concentrations of other fermentable sugars, furfural, hydroxymethylfurfural, acetic acid, and phenolic compounds were not determined. Although glucose, arabinose, and inhibitory compounds were not quantified in the present corn cob hydrolysate, previous studies have demonstrated that lignocellulosic hydrolysates contain a mixture of fermentable sugars in addition to xylose. Experimental investigations on corn cob hydrolysates reported the concentrations of glucose and arabinose to be around 5–10 and 1–5 g/L, respectively, particularly under mild dilute-acid pretreatment conditions.^39–42^ Alongside fermentable sugars, acid and thermal pretreatments generate inhibitory byproducts. Common inhibitors include acetic acid, furfural, and hydroxymethylfurfural. The reported concentrations in corn-based and similar lignocellulosic hydrolysates vary depending on the pretreatment severity. Acetic acid is frequently detected at levels of approximately 1–4 g/L, while furfural and hydroxymethylfurfural are generally observed in the range of 0.03–0.8 g/L. These ranges are widely documented in the literature and reflect typical compositional profiles of acid-pretreated lignocellulosic biomass hydrolysates.^39,43–45^

### Isolation and Screening of Xylose-Assimilating Yeasts

Various naturally fermented pickle samples were collected and brought to the laboratory under aseptic conditions and used as an isolation source for xylose-assimilating yeasts. Isolation studies on YPX agar plates yielded 16 different fungal isolates based on their colony morphology. The isolates were then screened for their ability to produce xylitol using CCH. According to HPLC analysis, strain T12 showed the highest xylitol accumulation and was selected for further experiments. Subsequently, molecular identification of the strain T12 was carried out based on ITS gene sequence analysis. Based on the sequencing of the ITS gene region, the isolate T12 showed a 99% similarity to *P. fermentans* (GenBank number: PV876729). The evolutionary relationship of T12 was evaluated using the neighbor-joining method.^46^ This study is the first to document the utilization of corn cob by *P. fermentans* for xylitol production.

### Optimization of Culture Conditions for Xylitol Production

Nutritional and physicochemical factors are widely recognized for their effect on the product yield in microbial fermentation processes.^47^ It has been documented that microbial xylitol production is affected by several parameters, such as the substrate concentration, temperature, pH, inoculum size, and incubation time.^19,48^ Therefore, the current study aimed to optimize certain culture parameters in order to enhance xylitol production by *P. fermentans* T12. Preliminary experiments focused on determining the optimum temperature and pH for xylitol production. First, the optimal temperature for xylitol fermentation by strain T12 was determined. The experimental results revealed that maximum xylitol production occurred at 30 °C (6.08 g/L). Similar optimal temperatures for xylitol production have also been reported in the literature for studies performed using *P. fermentans*.^15,17,18^ To examine the impact of the initial pH of the culture medium on the metabolite yield during xylitol production, fermentation experiments were conducted using media with varying pH levels. An increase in xylitol accumulation was observed as the initial pH increased from 5 to 6, with the highest production of 6.54 g/L achieved at pH 6 (Table 1).

**Table 1:** Effect of Culture Parameters on Xylitol Production

**culture parameter**	**level**	**xylitol production** (g/L)
temperature (°C)	26	5.24
	28	5.56
	30	6.08
	32	5.72
	34	5.48
pH	5.0	5.64
	5.5	6.08
	6.0	6.54
	6.5	6.32
	7.0	6.15
CCH (mL/L)	50	6.54
	75	8.70
	100	10.2
	125	12.7
	150	11.3
yeast extract (g/L)	0.5	11.3
	1.0	12.7
	1.5	13.3
	2.0	14.1
	2.5	13.4
inoculum concentration (%)	1	11.8
	3	14.1
	5	15.6
	7	17.1
	9	16.2

The addition of commercial carbon sources, such as glucose, xylose, sucrose, and lactose, to culture media significantly influences the overall process costs. Therefore, organic wastes or industrial byproducts are often favored over refined carbon sources in microbial production processes to reduce expenses.^49,50^ In this context, lignocellulosic wastes are highly important in microbial xylitol production due to their abundance, renewability, and low cost as a source of fermentable sugars, especially xylose. Hemicellulose, a key fraction of lignocellulosic biomass, contains high levels of xylose, which is the main carbon source for xylitol-producing microorganisms.^9,51^ The current study aimed to produce xylitol using *P. fermentans* T12 with nondetoxified CCH. The effect of CCH at different concentrations (50–150 mL/L) was investigated. The results revealed that xylitol accumulation increased at higher CCH concentrations, and the highest xylitol concentration was obtained at 125 mL/L CCH with a yield of 12.7 g/L (Table 1). However, higher concentrations of CCH resulted in lower xylitol accumulation since increasing concentrations of sugar monomers adversely impacted microbial growth and product yield. This can also be attributed to the increasing concentrations of certain inhibitory compounds, such as furfurals or phenolics, in the CCH.

Xylitol production from xylose is strongly influenced by the intracellular redox balance and oxygen availability. Once transported into the cell through specific membrane transporters, xylose is reduced to xylitol by xylose reductase (XR), which predominantly uses NADPH as a cofactor. Xylitol may then be further oxidized to xylulose by xylitol dehydrogenase (XDH), which is an enzyme that depends on NAD^+^. The different cofactor requirements of XR (NADPH-dependent) and XDH (NAD^+^-dependent) create a redox imbalance during xylose metabolism. When NADPH is sufficiently available but NAD^+^ regeneration is restricted, the oxidation of xylitol is limited, resulting in its accumulation. Oxygen availability is a key factor in maintaining redox balance. Under oxygen-limited or microaerobic conditions, reduced NAD^+^ regeneration diminishes XDH activity, thereby promoting xylitol accumulation. In addition, elevated xylose concentrations in the substrate can enhance flux through XR, thereby increasing cellular NADPH consumption. This greater cofactor demand may further disrupt redox balance, particularly when oxygen limitation constrains NAD^+^ regeneration. Therefore, the enhanced xylitol production observed under microaerobic conditions and elevated substrate levels can be attributed to the combined effects of cofactor imbalance, restricted NAD^+^ regeneration, and sustained NADPH-dependent XR activity.^52–54^

Nitrogen sources also play a critical role in microbial fermentation processes. Among them, organic nitrogen sources are reported to enhance xylitol yield since they support biomass formation and increase xylitol biosynthesis.^55^ In this regard, the impact of different concentrations of yeast extract on xylitol production was investigated. As shown in Figure 1d, a yeast extract concentration of 2 g/L resulted in the highest xylitol accumulation (14.1 g/L) and was selected as the optimal value (Table 1).

**Figure 1:**
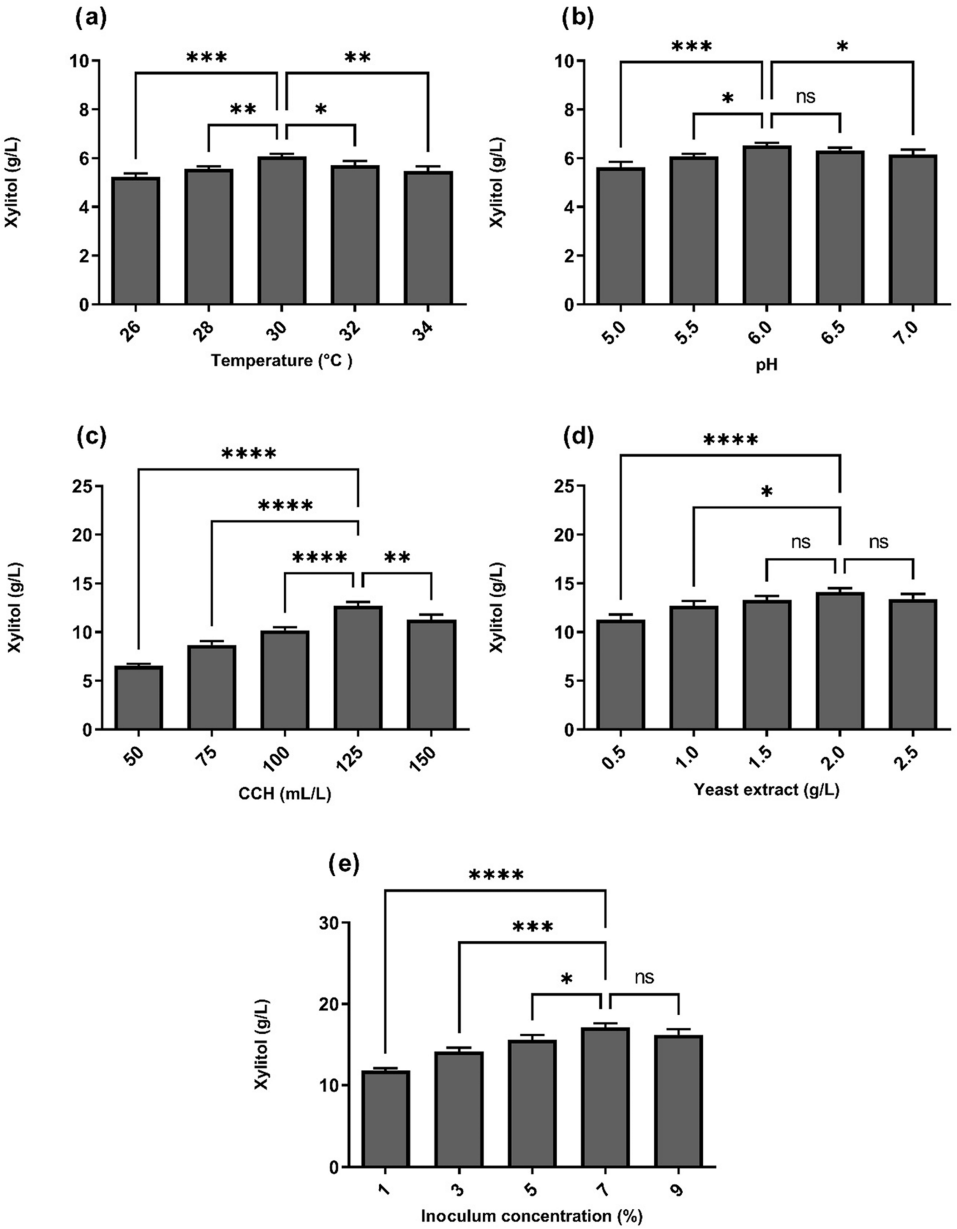
Effects of culture parameters on xylitol production by the strain T12: (a) temperature, (b) pH, (c) CCH concentration, (d) yeast extract concentration, and (e) inoculum concentration. Statistically significant changes are indicated by an asterisk (*). The symbols are as follows: *P < 0.05 (significant); **P < 0.01 (very significant); ***P < 0.001, and ****P < 0.0001 (extremely significant).

Optimization of the inoculum concentration is another key factor for achieving maximum metabolite yield. An appropriate inoculum level ensures a sufficient number of metabolically active cells at the start of fermentation, facilitating rapid adaptation and growth, thereby improving xylitol production. In contrast, excessively high inoculum levels cause intense competition for nutrients, which suppresses microbial growth and ultimately lowers product yield.^19,56^ As shown in Figure 1e, increasing the inoculum concentration enhanced xylitol accumulation up to an optimal point, beyond which further increases resulted in a gradual decrease in yield. Overall, an inoculum concentration of 7% was designated as the optimal value, resulting in a maximum xylitol yield of 17.1 g/L.

In the final phase of the optimization study, the effect of incubation time on xylitol production was evaluated. Xylitol production increased with the incubation time and reached a maximum at 96 h, after which prolonged incubation resulted in a decline in the product yield (Figure 2). Under the optimized culture conditions, *P. fermentans* T12 produced 21.6 g/L xylitol from 125 mL/L CCH (corresponding to 32 g/L xylose), achieving a yield of 0.67 g/g. The xylitol yield obtained in the present study is comparable to, and in some cases higher than, the values reported for other lignocellulosic hydrolysate-based fermentations. For example, Prabhu et al. reported 79.0 g/L xylitol with a yield of 0.54 g/g by an EMS mutant *P. fermentans* using Nondetoxified Sugarcane Bagasse Prehydrolyzate.^17^ Similarly, Narisetty et al. achieved higher xylitol titers during the fed-batch cultivation of *P. fermentans* using sugarcane bagasse and olive pit hydrolysates, reaching 86.6 and 71.9 g/L with yields of 0.75 and 0.74 g/g, respectively.^16^ In Corn cob-Based Processes, *Candida tropicalis* HDY-02 produced 58 g/L xylitol with a yield of 0.73 g/g under continuous fed-batch conditions, while *Pichia caribbica* produced 124.1 g/L xylitol with a yield of 0.80 g/g from detoxified and concentrated corn cob hydrolysate.^57,58^ Although previous studies reported higher xylitol titers, they generally employed fed-batch systems, detoxification steps, or engineered strains. In contrast, the present study achieved a relatively high xylitol yield under comparatively simple batch fermentation conditions using nondetoxified corncob hydrolysate, indicating that *P. fermentans* T12 has strong potential for sustainable and cost-effective xylitol production from agricultural residues.

**Figure 2:**
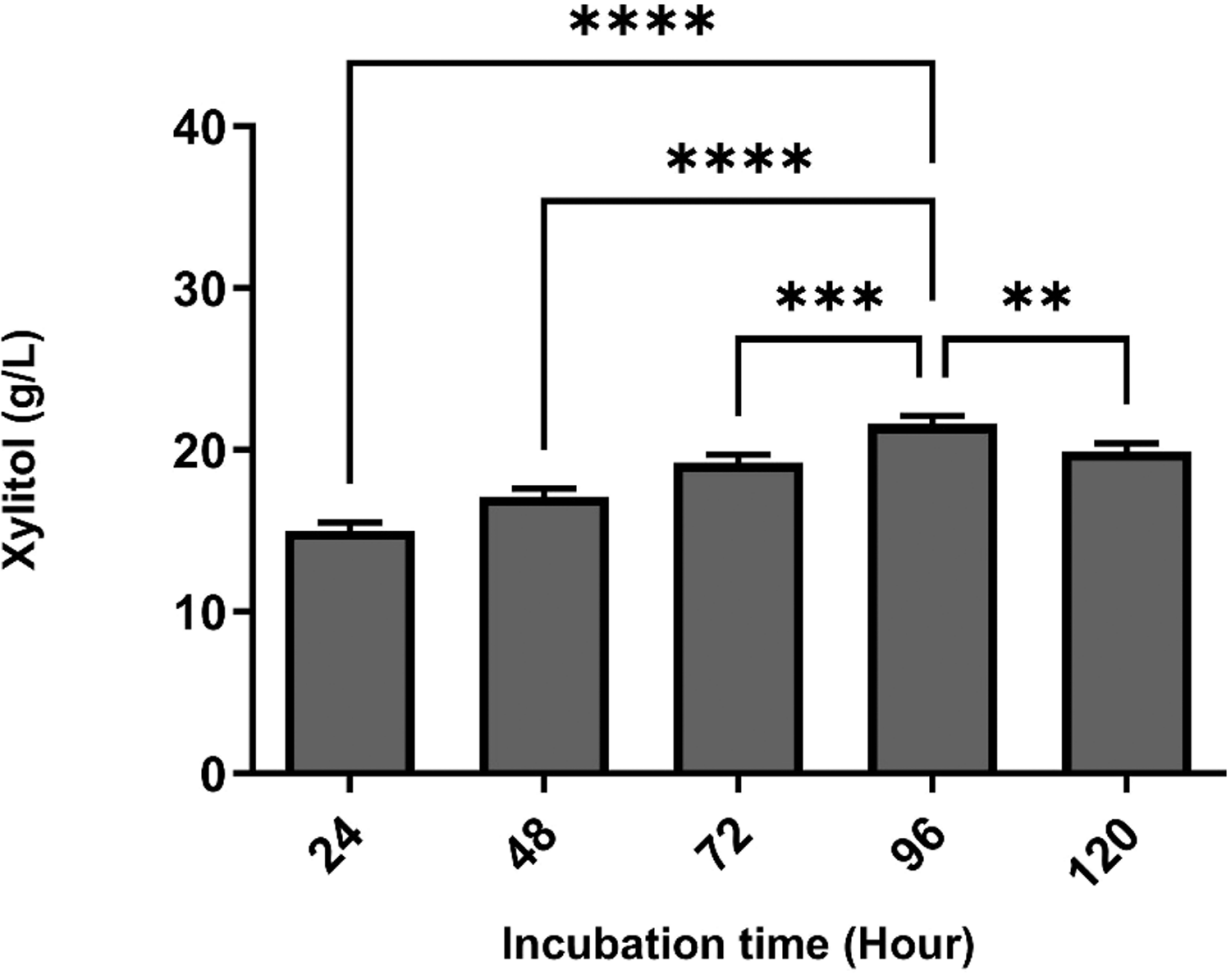
Kinetic experiments on xylitol production. Culture conditions: 30 °C, initial pH of 6.0, 125 mL/L CCH, 2 g/L yeast extract, and 7% inoculum concentration. Statistically significant changes are indicated by an asterisk (*). The symbols are as follows: *P < 0.05 (significant); **P < 0.01 (very significant); ***P < 0.001; and ****P < 0.0001 (extremely significant).

Moreover, FTIR spectroscopy confirmed the presence of the characteristic functional groups of microbial xylitol, demonstrating the successful formation of the crystalline structure.^19,59,60^ The FTIR spectra of both standard and microbial xylitol exhibit a broad O–H stretching band in the 3600–3200 cm^–1^ range typical of polyhydric alcohols, along with aliphatic C–H stretching vibrations at 3000–2850 cm^–1^, O–H bending, and CH_2_ deformation bands between 1650 and 1350 cm^–1^, and intense C–O stretching bands in the 1200–1000 cm^–1^ region, thereby confirming the xylitol structure. However, slight variations were observed in the band intensity and sharpness, especially in the O–H and C–O regions, which can be attributed to variations in hydrogen bonding and crystallinity rather than the formation of new functional groups (Figure 3).

**Figure 3:**
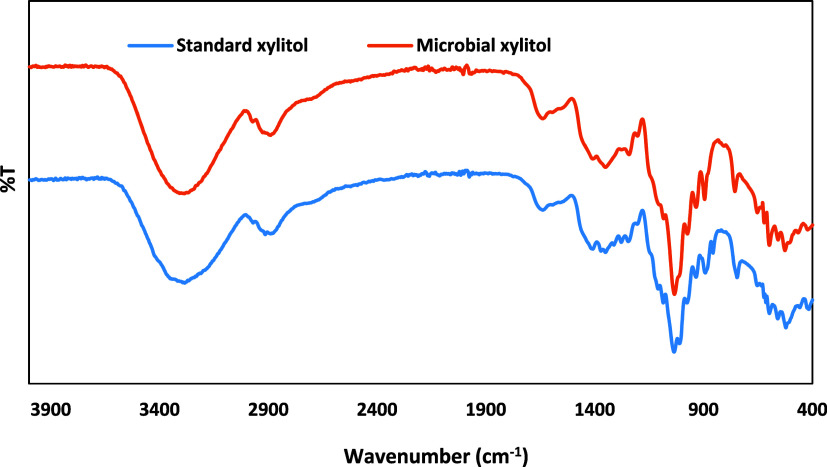
FTIR chromatogram of standard and microbial xylitol, showing peaks corresponding to the functional groups.

## Conclusion

This study investigated the ability of a newly isolated yeast strain, *P. fermentans* T12, to assimilate xylose and produce xylitol from agricultural waste. Corn cobs represent promising raw materials for green energy and bioproduct synthesis. Following acid pretreatment, the hemicellulosic hydrolysate derived from corn cobs can be directly used for xylitol fermentation without detoxification, achieving favorable xylose conversion and economic viability. In contrast to conventional methods that depend on refined substrates, this approach highlights corn cobs as a sustainable and cost-effective feedstock for *P. fermentans*, transforming an abundant industrial byproduct into value-added compounds. A key advantage of strain T12 is its capacity to produce xylitol from nondetoxified hydrolysates, which enhances process efficiency and environmental sustainability. Future studies should focus on scaling the process from laboratory systems to pilot-scale operations, conducting techno-economic analyses, and evaluating overall environmental impacts.

## Supplementary Material



## Notes

HPLC chromatograms, phylogenetic tree, and process flowchart (Figures S1–S4) (PDF)
