# Supplementary material for: Biovalorization of Nondetoxified Corn Cob Hydrolysate into Xylitol by Pichia fermentans T12
Source: ACS Omega. 2026 Jun 18;11(25):36908–15. doi: 10.1021/acsomega.6c00635 (PMC13325169; doi:10.1021/acsomega.6c00635)
Supplement: Supplementary file 1 [file ao6c00635_si_001.pdf]

# Supporting Information

## Biovalorization of non-detoxified corn cob hydrolysate into xylitol by *Pichia fermentans* T12

**Mehmet Akif Omeroglu**[1*]

[1]Department of Molecular Biology and Genetics, Faculty of Science, Ataturk University, Erzurum, Turkey

***Corresponding author:** akif.omeroglu@atauni.edu.tr

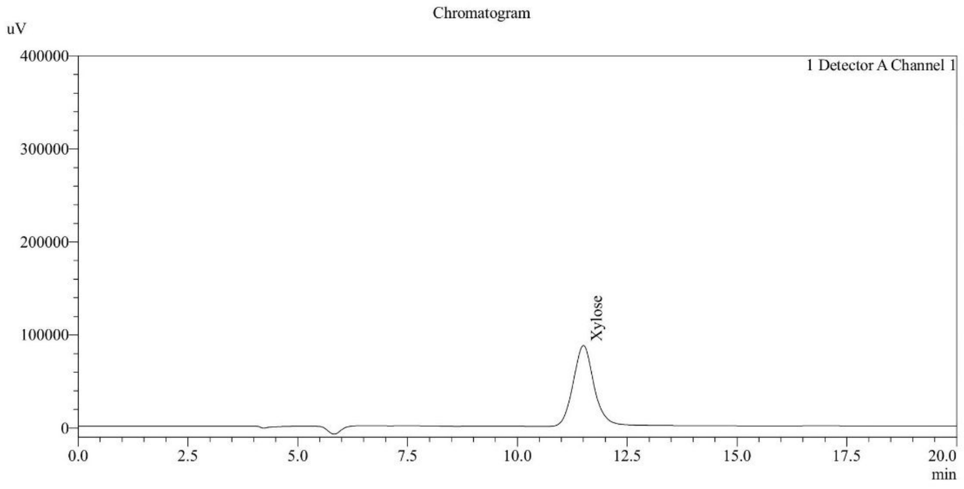


**Figure S1.** HPLC chromatogram of raw CCH, showing its xylose content (Retention time of 11.506).

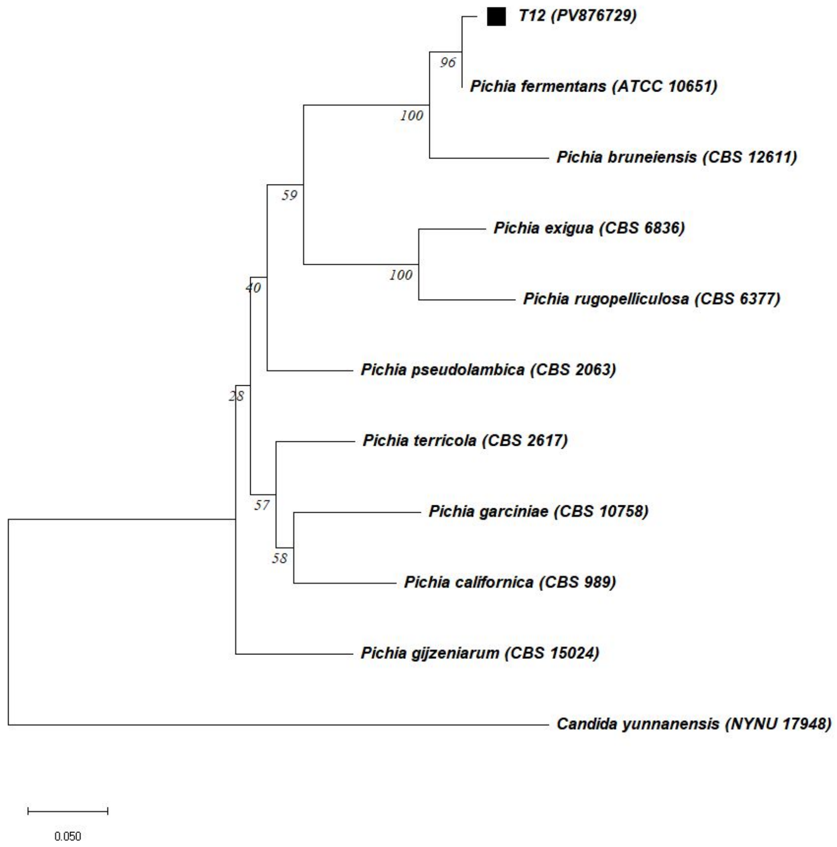


**Figure S2.** Phylogenetic tree of the isolate T12 on the basis of ITS gene sequence. Tree was constructed by a neighbor-joining method. Bootstrap values were based on 100 replicates. *Candida yunnanensis* was used as out-group. The scale bar represents 0.05 changes per nucleotide position.

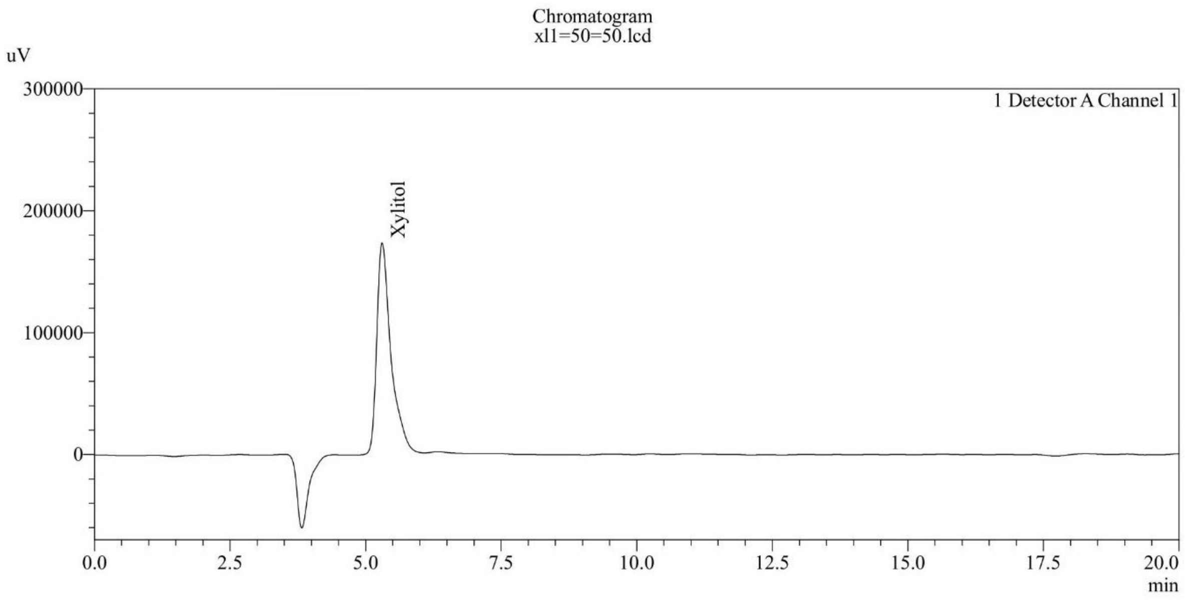


**Figure S3.** HPLC chromatogram of microbial xylitol under optimized culture conditions (Retention time of 5.306).

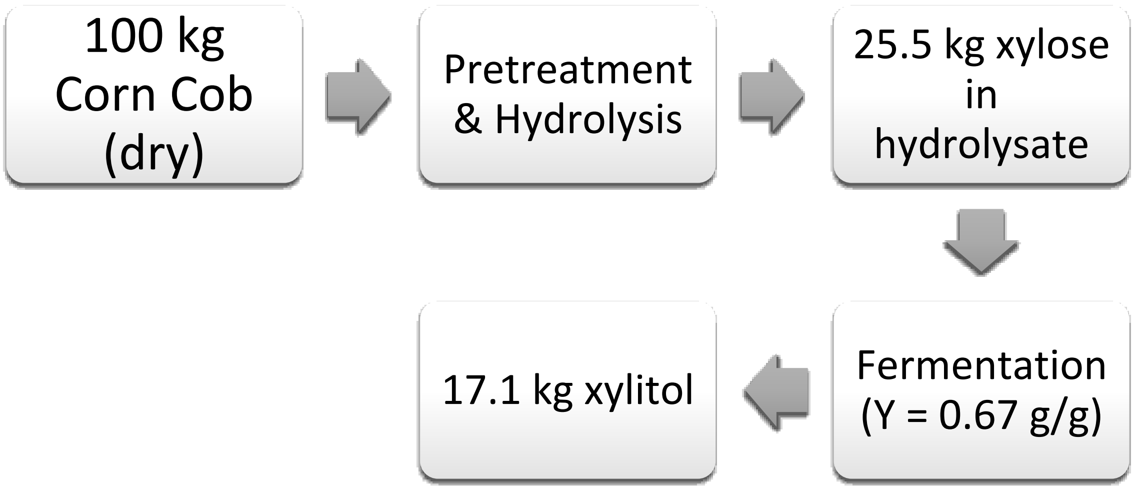


**Figure S4.** Process flowchart for xylitol production from corn cob biomass.
